# Eosinophilic Gastroenteritis Presenting As Unexplained Chronic Abdominal Pain

**DOI:** 10.7759/cureus.8640

**Published:** 2020-06-15

**Authors:** Salem Gaballa, Kyaw M Hlaing, Nathan Mahler, Richard Hargrove, Marigny Roberts

**Affiliations:** 1 Internal Medicine, LewisGale Medical Center, Salem, USA; 2 Pathology, LewisGale Medical Center, Salem, USA

**Keywords:** eosinophilic gastroenteritis, chronic abdominal pain, endoscopy, git endoscopy, eosinophilic mucosal infiltration, egg allergy

## Abstract

A 27-year-old Caucasian female was hospitalized three times over a four-month period for recurrent, intermittent abdominal pain associated with nausea and diarrhea. No signs or symptoms of gastrointestinal (GI) bleeding were present. A stool occult blood test and stool enteric pathogen tests were negative. A complete blood count (CBC) revealed a peripheral blood eosinophil count of 1080 cells /µL without any inflammatory reaction. An upper endoscopy showed grossly normal-appearing esophageal and duodenal mucosa; however, a gastric mucosal biopsy showed an eosinophil infiltration of ≥20 eosinophils/high power field (HPF). Based on these findings, she was diagnosed with eosinophilic gastroenteritis (EGE). A definitive diagnosis of EGE should be confirmed with both an analysis of gastrointestinal mucosal biopsy and an elevated peripheral blood eosinophil count. Specifically, histological evaluation of the mucosal tissue must show an eosinophilic infiltration rate of 20 eosinophils/HPF. The diagnosis should be followed by an extensive review of the patient’s allergic disease history.

## Introduction

Eosinophilic gastroenteritis (EGE) is a rare disorder that is characterized by eosinophil infiltration of the gastrointestinal (GI) tract, excluding the esophagus. The symptoms are usually nonspecific such as chronic abdominal pain, mild to moderate steatorrhea, failure to thrive, lower gastrointestinal bleeding, and anemia. The disease can present in combination with eosinophil accumulation in the pancreas and idiopathic hyper-eosinophilic syndrome (HIS). EGE should be diagnosed based on gastrointestinal mucosa pathology. We present a case of chronic recurrent abdominal pain secondary to eosinophilic gastroenteritis induced by food allergies.

## Case presentation

A 27-year-old Caucasian female with a medical history of hypertension, bipolar disorder, and food allergies presented to the emergency department with complaints of right upper quadrant abdominal pain for the last three months. The pain was described as dull and cramping in nature, aggravated by greasy meals, associated with nausea and loose stools, and alleviated with bismuth subsalicylate. The patient endorsed decreased appetite but no weight change. The patient reported occasional alcohol use and smoking 10 cigarettes daily. History of methamphetamine use was reported, but she had been abstinent for 70 days. The patient's family history was positive for pancreatic cancer (mother) but no other cancers or autoimmune diseases.

On physical examination, her vital signs were normal. Heart and lung sounds were normal. Abdominal examination revealed diffuse abdominal tenderness without rigidity or guarding. No palpable masses or visceromegaly were appreciated. Bowel sounds were normal, and there were no visible rashes or lesions on skin examination.

Laboratory results showed the following: hemoglobin 14.2 g/dL, leukocytes 18,350/mm^3^ (neutrophil: 25%, eosinophil: 61%, lymphocyte: 14%), platelet count 256,000 uL. The complete metabolic profile was within the normal range. Lipase was 2300 u/L, amylase was 28 u/L, and lipid panel was within the normal range, procalcitonin was <0.05 ng/mL, and blood alcohol level was < 50 mg/dL (normal acceptable range 0-50 mg/dL or 0%-0.05%). A urine drug screen was negative. Blood cultures showed no growth after five days. Serum immunoglobulins were within a normal range, including a normal immunoglobulin G4 (IgG4) level. Celiac disease antibodies were negative. Parasitological examination and bacterial culture of the stool were normal. Ultrasound of the right upper quadrant showed a fatty liver with a normal gallbladder. Computed tomography (CT) of the abdomen with intravenous (IV) contrast showed a normal-appearing pancreas, and a hepatobiliary scintigraphy (HIDA) scan showed normal gallbladder function. The diagnosis of hyper-eosinophilic syndrome was excluded based on the absence of organ damage and an otherwise normal hematologic workup.

**Figure 1:**
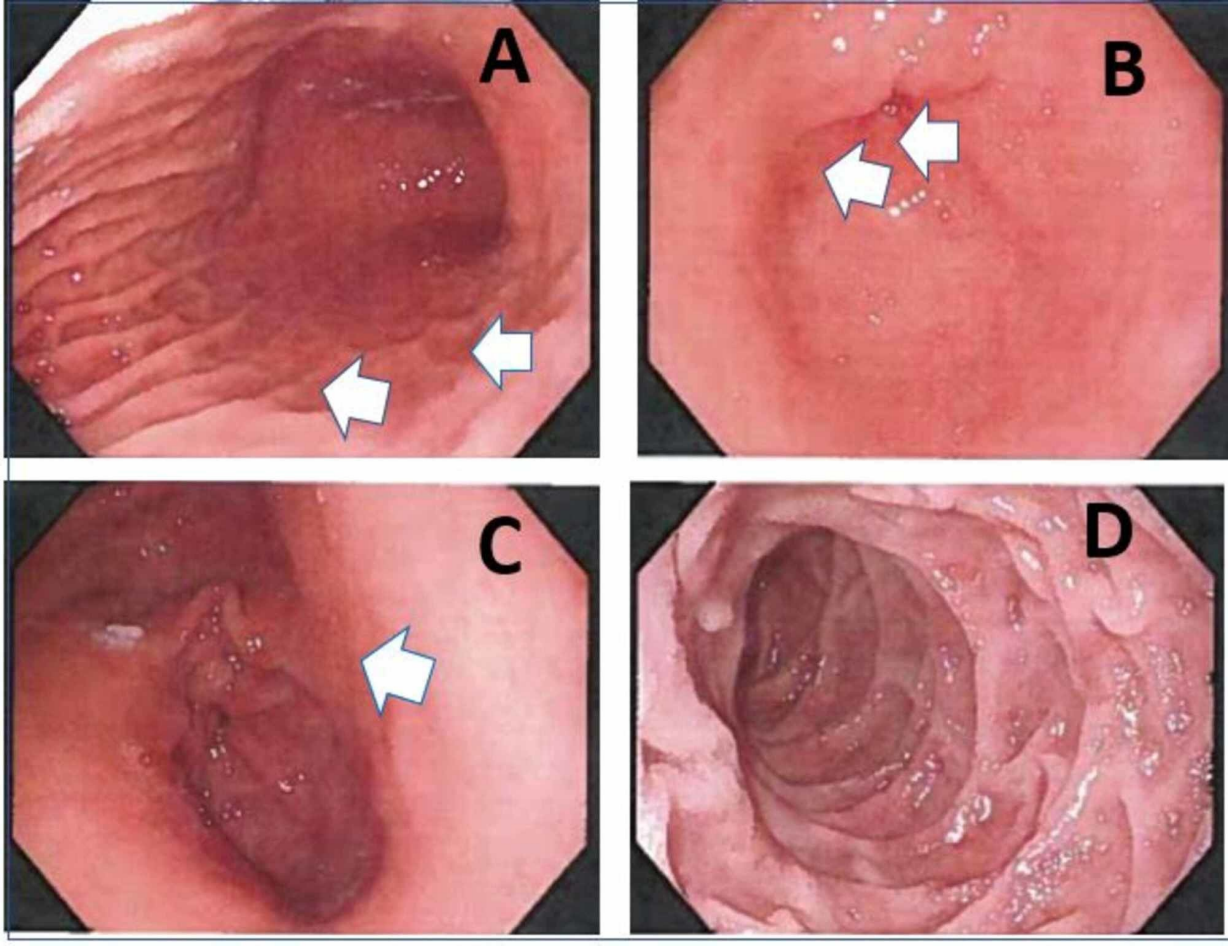
Findings from upper endoscopy ( as indicated by the white arrow) A) Gastric fundus showing moderate gastritis. B) Gastric antrum showing moderate gastritis. C) Gastric pylorus showing pyloric sphincter with gastritis. D) The second segment of the duodenum showing no inflammatory changes

**Figure 2:**
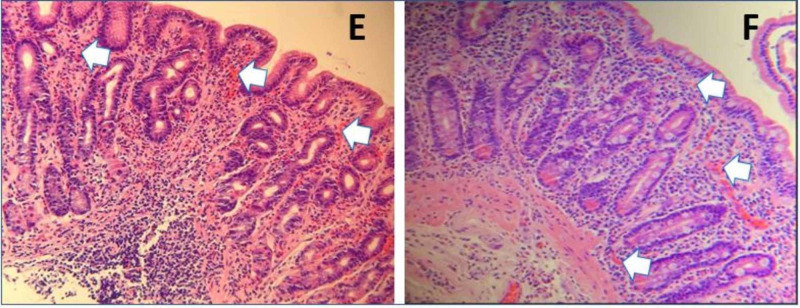
Histopathology images (Hematoxylin and Eosin ×400) E) Gastric pathology showing mucosa with mild superficial inflammation in the lamina propria. The inflammatory cells consist almost entirely of eosinophils (≥20 eosinophils/HPF), some of which are degranulating. F) Duodenal pathology showing mucosa with unremarkable villous architecture without villous blunting. The lamina propria contains a moderate amount of chronic inflammation consisting of plasma cells, lymphocytes, and increased numbers of eosinophils(≥20 eosinophils/HPF). In areas, the eosinophils are the main inflammatory cell component. Infectious organisms are not identified.

Upper gastrointestinal endoscopy (Figure 1) revealed a grossly normal endoscopic mucosa from the esophagus to the second part of the duodenum except for some superficial mucosal inflammation of gastric mucosa. The mucosal biopsy (Figure 2) showed eosinophilic infiltration (≥20 eosinophils/high power field (HPF)) from the gastric body to the duodenal bulb. There was no eosinophilic infiltration of the esophagus. Based on the above findings, she was diagnosed with EGE and treated with prednisolone therapy at a dose of 30 mg twice daily. A few days after the initiation of therapy, symptoms improved, and steroid therapy was maintained for two weeks. After the cessation of the therapy, the eosinophil count had dropped significantly. She also started the six-food elimination diet (SFED), which trials the exclusion of wheat, milk, egg, nuts, soy, fish, and shellfish, and has been associated with better outcomes in these patients. After two weeks, she experienced significant clinical improvement while on steroid treatment. However, the eosinophil count rebounded after completing treatment, indicating a possible need for long-term steroid treatment. She was also referred to an allergist.

## Discussion

EGE includes a group of disorders characterized by the inflammation of various organs with eosinophilic infiltration. Up to 50% of patients with eosinophilic gastroenteritis give a history of allergic diseases such as asthma, rhinitis, drug and food allergies, and eczema [1]. Eosinophilic disorders affecting the GI tract below the esophagus are more severe and harder to treat. Only a small percentage of patients, if any, respond to dietary treatment. Oral corticosteroids are often essential to induce remission [2]. The inflammation is caused by eosinophilic infiltration and degranulation. Interleukin-3 (IL-3), IL-5, and granulocyte-macrophage colony-stimulating factor (GM-CSF) may be responsible for the recruitment and activation of eosinophils [3]. It is proposed that food allergens across the intestinal mucosa and trigger an inflammatory response that includes mast cell degranulation and the recruitment of eosinophils [4]. Per Nomura et al., IL-33 and thymic stromal lymphopoietin (TSLP) were significantly elevated in patients with EGE and their concentrations correlate with EGE activity. In addition, the levels of IL-33 and TSLP decreased and the symptoms were improved by the elimination of food allergens from the diet [5].

Four criteria of eosinophilic gastroenteritis are the presence of gastrointestinal symptoms, eosinophilic infiltration of the GI tract, exclusion of parasitic disease, and absence of other systemic involvement. Symptoms vary depending on the involved organ and the extent of involvement such as the stomach, the duodenum, the colon, or multiple locations in the GI tract. If eosinophils infiltrate the muscular layer, there can be obstruction or perforation. Subserosal involvement is associated with eosinophilic ascites. Peripheral eosinophilia is present in the majority of patients with EGE. The differential diagnosis of Hypereosinophilic syndrome (HES) should be considered in cases with moderate‐to‐severe eosinophilia (>1500 eos/mm^3^); however, it can be ruled out when there is an absence of eosinophilic infiltration in all other organs except the bowel [6-8].

Eosinophilic gastrointestinal disease (EGID) can be associated with abdominal pain, bloating, and gastrointestinal bleeding. Patients with gastric inflammation typically have nausea, vomiting, and early satiety. Those with duodenal involvement have malabsorption and protein‐loss enteropathy. Colonic involvement is associated with diarrhea. Unlike isolated eosinophilic esophagitis (EOE), EGID is rarely associated with strictures in any organ [8]. Biopsies should be obtained from five to six different sites per involved segment (e.g., stomach, duodenum), and the gross appearance may be normal on endoscopy. Eosinophils in ascites fluid can also be diagnostic. The presence of pseudo-polyps or eosinophils in particular tissue layers (i.e., intraglandular eosinophils or in the muscular layer), conglomerates of eosinophils in an abscess, or eosinophilic sheets in the lamina propria make the diagnosis of EGID more likely. Food allergens play a heavy role in multi-organ and colonic disease and have at least minor involvement in all forms of EGID [9]. A recent study addressed the question of whether EOE and non‐EOE‐EGID are a spectrum of the same disease or if they should be qualified as distinct disease entities. Caldwell et al. analyzed genome‐wide transcript profiles and concluded that non‐EOE‐EGID is a systemic disorder, and only 7% of patients with non‐EOE‐EGID had a transcriptome that overlapped in patients with EOE [10].

Most patients are treated initially with systemic corticosteroids (0.5‐1 mg/kg/d for five to 14 days), which have a reported response rate of up to 90%. Once symptoms are controlled, corticosteroids are typically tapered over two to four weeks. The appropriate duration of steroid treatment is unknown and relapse often necessitates long-term treatment. Dietary adjustment is effective in 25%‐60% of patients with gastric involvement and includes an elemental or six‐food elimination diet. Swallowed budesonide has been reported to be effective in non‐EOE‐EGID [11]. Budesonide is a corticosteroid with high first‐pass metabolism. Enteric‐coated capsules have been designed to release the drug as distally as the ileum. Previously, the "triple‐phase" enteric‐targeted budesonide capsule has been described for use in diffuse enteropathy [11]. Manipulation of the capsule prior to ingestion targets the gastric and proximal small intestinal mucosa with a topical corticosteroid, thus avoiding systemic immunosuppression. Specifically, this entails ingesting a combination of crushed, opened, or intact enteric‐coated budesonide capsules. Crushing the capsule bypasses both the time‐ and pH‐dependent release of the medication, permitting release into the stomach. The opened capsule releases into the upper small intestine. The whole, unopened capsule targets the distal small intestine. Monoclonal anti‐IL‐5, reslizumab, has been used in an open‐label clinical trial of four patients [12], in which it was very effective in decreasing peripheral and gastric eosinophilia. The monoclonal antibody (anti‐IgE) omalizumab was not effective in treating tissue eosinophilia. Other anti-allergic drugs have been trialed with varying results. Montelukast and sodium cromoglycate have shown inconsistent results on sustaining remission of EGE. Cases with an associated food allergy can be treated using an elimination diet. Spontaneous recovery after fasting for several days has also been established [13].

## Conclusions

In summary, EGE is a rare disease with varied symptom presentation and a good prognosis. It is seen in patients of any age or race and can present with multiple complications, including malnutrition, obstruction, and intestinal perforation. A mucosal eosinophilic infiltration (> 20 eosinophils/HPF) is the key for correct diagnosis even in a grossly normal-appearing mucosa. Corticosteroid treatment is the mainstay therapy for the induction of remission of EGE, with long-term therapy required in some cases.
